# Acetylation proteomics and metabolomics analyses reveal the involvement of starch synthase undergoing acetylation modification during UV-B stress resistance in *Rhododendron Chrysanthum* Pall

**DOI:** 10.1186/s41065-024-00320-4

**Published:** 2024-05-03

**Authors:** Meiqi Liu, Li Sun, Yuhang Cao, Hongwei Xu, Xiaofu Zhou

**Affiliations:** 1 Jilin Provincial Key Laboratory of Plant Resource Science and Green Production, Jilin Normal University, Siping, China; 2 Siping Central People’s Hospital, Siping, China

**Keywords:** *R. Chrysanthum*, Starch and sucrose metabolism, UV-B stress, Acetylated proteome, Metabolome

## Abstract

**Background:**

*Rhododendron chrysanthum* Pall. (*R. chrysanthum*) is a plant that lives in high mountain with strong UV-B radiation, so *R. chrysanthum* possess resistance to UV-B radiation. The process of stress resistance in plants is closely related to metabolism. Lysine acetylation is an important post-translational modification, and this modification process is involved in a variety of biological processes, and affected the expression of enzymes in metabolic processes. However, little is known about acetylation proteomics during UV-B stress resistance in *R. chrysanthum*.

**Results:**

In this study, *R. chrysanthum* OJIP curves indicated that UV-B stress damaged the receptor side of the PSII reaction center, with a decrease in photosynthesis, a decrease in sucrose content and an increase in starch content. A total of 807 differentially expressed proteins, 685 differentially acetylated proteins and 945 acetylation sites were identified by quantitative proteomic and acetylation modification histological analysis. According to COG and subcellular location analyses, DEPs with post-translational modification of proteins and carbohydrate metabolism had important roles in resistance to UV-B stress and DEPs were concentrated in chloroplasts. KEGG analyses showed that DEPs were enriched in starch and sucrose metabolic pathways. Analysis of acetylation modification histology showed that the enzymes in the starch and sucrose metabolic pathways underwent acetylation modification and the modification levels were up-regulated. Further analysis showed that only GBSS and SSGBSS changed to DEPs after undergoing acetylation modification. Metabolomics analyses showed that the metabolite content of starch and sucrose metabolism in *R. chrysanthum* under UV-B stress.

**Conclusions:**

Decreased photosynthesis in *R. chrysanthum* under UV-B stress, which in turn affects starch and sucrose metabolism. In starch synthesis, GBSS undergoes acetylation modification and the level is upregulated, promotes starch synthesis, making *R. chrysanthum* resistant to UV-B stress.

**Supplementary Information:**

The online version contains supplementary material available at 10.1186/s41065-024-00320-4.

## Introduction

Of all solar radiation reaching the surface of the earth, ultraviolet (UV) light accounts for 7% [10]. UV can be classified into three categories based on the wavelength range: UV-A, which has a wavelength range of 315–400 nm; UV-B, which has a wavelength range of 280–315 nm; and UV-C, which has a wavelength range of 100–280 nm [3]. Of the three types of UV radiation, UV-C has the highest energy and is completely absorbed by the ozone layer (O_3_). UV-A is also fully absorbed by the ozone layer [2]. However, only UV-B is not fully absorbed by the ozone layer, by approximately 95% absorption, and the remaining unabsorbed UV-B reaches the surface of the earth and has some effect on organisms. The average intensity of UV-B reaching the surface of earth has been reported to be 1 W.m^−2^ [4]. However, depending on the dose, UV-B can also cause some damage to organisms on the Earth’s surface. As a result of anthropogenic emissions, the natural and dynamic equilibrium of ozone has been disrupted, and ozone levels have declined, leading to an increase in UV-B radiation [37]. Although regulations banning global emissions of ozone-depleting substances have been subsequently enacted, few signs of ozone layer recovery have been reported [1].

The plant, due to its own fixation, encounters adverse environmental conditions causing abiotic or biotic stress during its lifespan. UV-B primarily targets, such as photosynthesis, proteins, DNA and membranes, to affect plant growth, development and physiological functions and ultimately the plant’s own adaptation to the environment [10, 11, 17]. Although much relevant and useful information related to UV-B tolerance has been accumulated from the analysis of UV-B resistance in plants such as *Arabidopsis thaliana* [27, 32, 34], the mechanisms of plant resistance to UV-B stress are extremely complex. Accordingly, many studies have utilized techniques such as proteomics, posttranslational modificationomics, and metabolomics to study UV-B radiation-tolerant plants, with the aim of exploring the molecular mechanisms of UV-B radiation tolerance in plants. As a plant growing in the alpine tundra zone, *R. chrysanthum* has evolved certain anti-UV-B radiation mechanisms during its own growth and development, which makes it a great and appropriate experimental material for researching the mechanisms of plant resistance to abiotic stresses. In prior studies, various aspects of the response processes of *R. chrysanthum* in the face of UV-B stress were investigated and analyzed, including photosynthesis [22], ABA signaling [39], amino acid metabolism [38], and the antioxidant system [46] in response to UV-B stress. In the preliminary stage, we used wild-type and domesticated *R. chrysanthum* as experimental materials and set different UV-B radiation doses. Finally, we determined that the optimal UV-B radiation dose was 48 h, and *R. chrysanthum*, the wild type, responded more clearly to UV-B, demonstrating a strong and fruitful countermeasure mechanism [25].

Starch and sucrose in the plant body play a vital role when subjected to UV-B stresses [38]. Starch is a type of carbohydrate and an essential storage form of carbohydrates, and starch synthesis occurs mainly in chloroplasts. The plant body synthesizes starch using triose phosphate produced by the Calvin cycle as a substrate in the presence of 1,4-alpha-glucan branching enzyme (GBE), granule-bound starch synthase (GBSS), starch synthase (SS), and starch debranching enzyme (DBE), while α-amylase [11] and β-amylase [20] are involved in starch catabolism. Plant bodies under conditions where light is available for photosynthesis undergo carbon fixation and starch synthesis in chloroplasts. Starch in this case can accumulate briefly in the chloroplasts as a temporary carbohydrate. This temporary carbohydrate deposition can provide the needed energy and carbon for the whole plant during the dark period, when the plant body is unable to photosynthesize or photosynthesis is inhibited [36]. Meanwhile, starch synthesis and degradation are closely related to sucrose metabolism. Sucrose is synthesized and degraded by sucrose synthase (SuS), invertase [8] and sucrose-phosphate synthase (SPS) using triose phosphate as a substrate [33]. In a previous study, we used metabolomics as an entry point to find that the metabolite contents and transcripts in the sucrose and starch metabolism pathway of *R. chrysanthum* remained consistent under UV-B stress [38].

In fact, plant defense against stress may produce more significant metabolic changes [15]. Metabolites are the basis of an organism’s life activity, and changes in metabolites can be used to visualize and clearly understand the biological processes of plant resistance to abiotic stresses. Over the last several years, studies on the resistance of plant bodies to adversity stress have made extensive use of proteomic and metabolomic data to analyze internal changes in organisms under adversity stress, to identify proteins or enzymes, metabolites and metabolic pathways of important pathways in the form of proteomics as the cause and metabolomics as the effect and to construct the core regulatory network to reveal the response of plants to abiotic stresses [7, 30, 31]. For instance, combined metabolomic and proteomic analyses explain that primary metabolism in *Pinus radiata* under UV light deals with oxidation caused by stress while reducing ROS production [30]. Proteomics and metabolomics data confirmed the promotional effect of methyl jasmonate on ganoderic acid (GA) biosynthesis in *Ganoderma Lucidum*  [18]. Proteomic and metabolomic analyses provided new insights into the complex mechanisms of chickpea (*Cicer arietinum* L.) response to drought stress by identifying changes in key enzymes and metabolites involved in isoflavone biosynthesis and galactose metabolism in chickpea [19]. Indeed, a great deal of research has focused on the proteome. However, when a plant is stressed, proteins in the plant undergo posttranslational modifications (PTMs) to adapt to the stress.

PTMs of proteins play an imperative and crucial role in plant resistance to abiotic stresses, lysine acetylation (Kac) is one of the major PTMs, and they are mainly used to regulate protein function in several ways, such as acetylation, which can alter protein activity, subcellular localization of proteins and protein stability [6, 29]. Today, a great deal of research is focused on histone Kac, which has been found to play crucial roles in biological processes in a wide range of organisms. Advances in proteomics have provided new ideas and insights into nonhistone Kac. Although there have been studies on nonhistone Kac in plants [9, 21, 23, 47], there are still few studies on plant stress tolerance. In a previous study, using lysine acetylation as an entry point, it was found that *R. chrysanthum* undergoes UV-B stress, photosynthesis is inhibited, and the photosystem is impaired, but the level of acetylation of the proteins of the photosystem is changed, which mitigates photodamage [22]. Previous studies have mainly focused on photosystem proteins and have not delved into the changes in acetylation modification levels and modification effects of pathway proteins related to photosynthesis. Because photosynthesis is inextricably linked to the utilization and distribution of plant body sugars, studies on photosynthesis in *R. chrysanthum* provide a certain research basis for starch and sucrose metabolism.

Therefore, in this research-based work, the acetylated proteome and broad targeting metabolome of *R. chrysanthum* leaves were analyzed under 48 h PAR + UV-B and 48 h PAR conditions using *R. chrysanthum* as the experimental material. By digging differentially expressed acetylated proteins (DAPs), these proteins can be further analyzed. To identify the enzymes, metabolites, and metabolic pathways related to starch and sucrose metabolism in *R. chrysanthum* and to systematical and comprehensive analyze the molecular mechanism of the *R. chrysanthum* response to UV-B. The results of the study, that is, the role of acetylation of key enzymes in the starch and sucrose metabolic pathways of *R. chrysanthum* in response to UV-B duress, will serve as favorable information for plant resistance to adversity and provide some basis for the cultivation of UV-B stress-resistant crops.

## Materials and methods

###  Plant material growth conditions

*R. chrysanthum* was obtained from the Key Laboratory of Plant Resource Science and Green Production, Jilin Province, China. The *R. chrysanthum* were placed in an artificial climate chamber for growth. The chamber was set up to simulate an alpine environment with the following settings: with light for 14 h and 18 °C. In the absence of light, the time was 10 h, and the temperature was 16 ℃. The relative humidity in the climate chamber was 60%.

### Experimental design

**Fig. 1:**
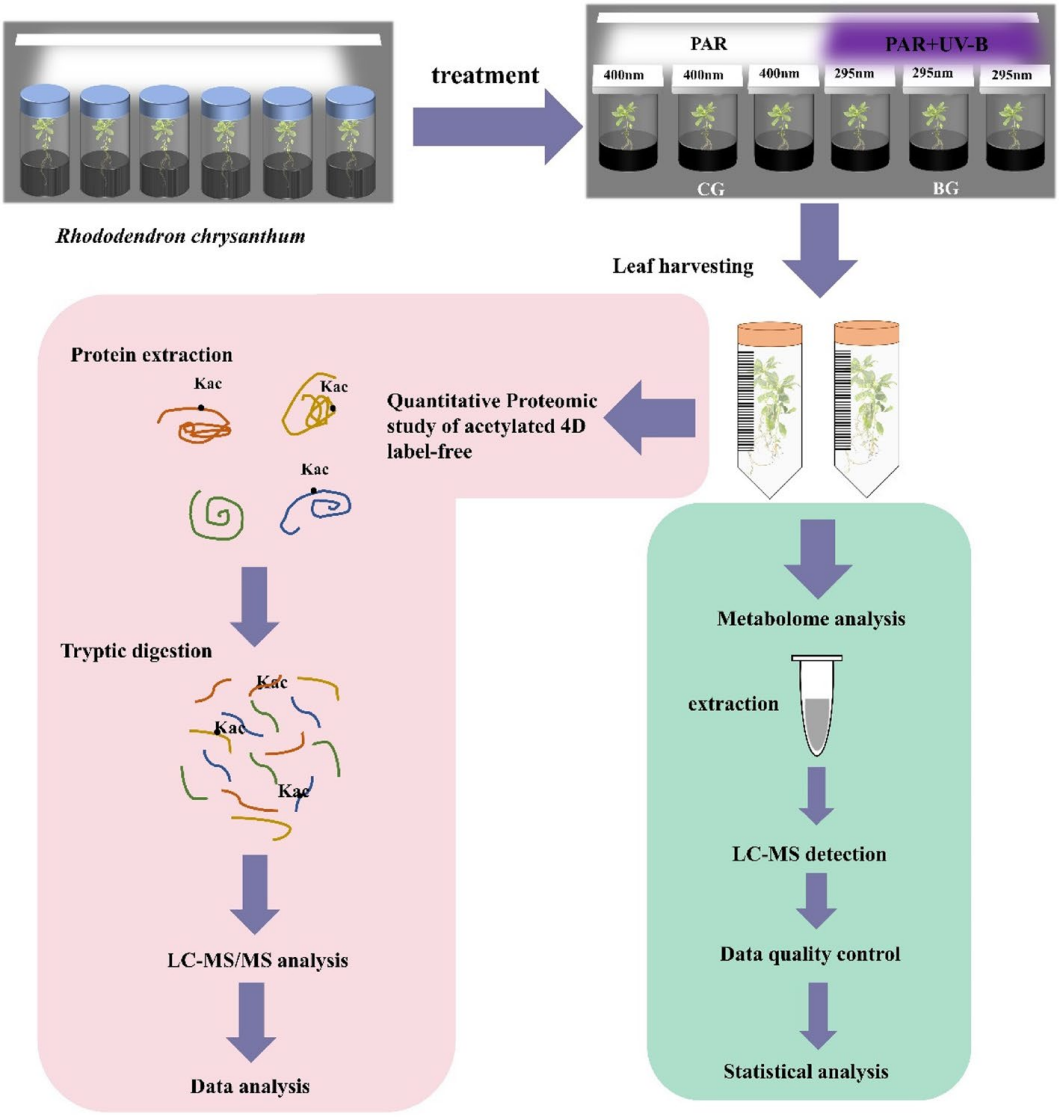
Process of UV-B radiation experimental treatment and acetylated proteome and metabolomics analysis in *R. chrysanthum*

*R. chrysanthum* was divided into two groups, and the experiment was repeated three times in each group. A group of *R. chrysanthum* was treated with PAR and recorded as the CG group; the other group was treated with UV-B and PAR and was recorded as the BG group. The time was 48 h. Subsequently, the treated plant material was placed into sampling tubes and sent to Hangzhou Jingjie Biotechnology Co. for quantitative proteome of acetylated 4D label-free and metabolomic testing and analysis (Fig. 1). By using longpass filters with different transmission characteristics as a means of achieving the desired two radiation conditions. A 400 nm filter (Edmund, Filter Long 2 in SQ, NJ, USA) was placed on the culture bottles of *R. chrysanthum* to be treated with PAR. For PAR + UV-B treatments, a 295 nm filter was placed above the *R. chrysanthum* culture bottles to allow radiation to pass through the filter to achieve PAR + UV-B treatment. During the radiation process, the bottles were wrapped in tinfoil to ensure that the radiation passed through the filters to the plants. During the radiation treatment, visible light, PAR, was provided by warm white fluorescent lamps (Philips, T5 × 14 W, The Netherlands). The artificial UV-B radiation source was realized by UV-B fluorescence tubes (Philips, Ultraviolet-B TL 20 W/01 RS, The Netherlands). Based on the transmission function of the longpass filters, the irradiance effectively received by the test material, *R. chrysanthum*, was 2.3 W m^–2^ UV-B, and PAR of 50 µmol (photon) m^–2^ s^–1^.

### OJIP transient measurements

Chlorophyll OJIP fluorescence-induced kinetic curves were measured using a Handy PEA Plant Efficiency Analyzer (England). Plants were acclimatized in the dark for 30 min before measuring the OJIP curves. The OJIP curves refer to the minimum fluorescence intensity when all PSII reaction centers (RCs) are open (O step), the intensity at 2 ms (J step), the intensity at 30 ms (I step), and the maximum intensity when all PSII RCs are closed (P step).

### Measurement of physiobiochemical parameters

#### Method for determination of sucrose and starch content

The sucrose contents were determined by the anthrone reagent method [40], and the starch content was determined by the acid hydrolysis method [45].

### Statistical analysis

Experimental and metabolomics data were analyzed using SPSS statistics 26 software via independent samples t test.

### Quantitative proteomic study of acetylated 4D label-free

#### Protein extraction

*R. chrysanthum* was ground and pulverized in liquid nitrogen, transferred to centrifuge tubes, and approached 3 times on ice in lysis buffer (8 M urea, 2 mM EDTA, 10 mM DTT, and 1% protease inhibitor cocktail) with the aid of a high-intensity ultrasound processor (Scientz, Ningbo, China). After centrifugation at 20,000 * g for 10 min at 4 °C, the supernatant was centrifuged for 3 min at 4 °C. Proteins in the supernatant were centrifuged for 3 min at 4 °C and then precipitated with 15% TCA for 4 h at -20 °C. The rest of the sediment was washed three times with acetone after chilling in a refrigerator. Finally, the proteins were redissolved in buffer consisting of 8 M urea and 100 mM TEAB, pH 8.0. For the purpose of gauging the protein concentration in the supernatant, it was determined using the 2-D Quantay Analysis Kit.

#### Trypsin digestion

At 56 °C, in 30 min, the protein solution was deacidized by 5 mM dithiothreitol and then by 11 mM iodiacetamide in the dark for 15 min, and then 100 mM TEAB was added to diluted protein samples when the urea concentration was less than 2 M. Finally, trypsin was added during the first digestion, and the trypsin to protein mass ratio was 1:50. The time was overnight. Finally, a second digestion was performed by adding trypsin at a ratio of 1:100 trypsin to the protein mass for 4 h.

#### LC‒MS/MS analysis and database search

Formic acid (solvent A) was used to solubilize the tryptic peptides, which were then mounted straightforwardly onto a self-restraint analytical column. In a UPLC 1000 EASY-nLC instrument running at 400 nL/min, the solvent B gradient increased from 6 to 23% (0.1% formic acid dissolved in 98% acetonitrile) in 26 min, decreased from 23 to 35% in 8 min, increased to 80% in 3 min, and maintained at 80%, and then the flow rate decreased for 3 min. After NSI was applied to the peptides, tandem mass spectrometry (MS/MS) was carried out using a Q Exactive TM Plus (Thermo) coupled to a UPLC system. In the presence of the conjugate, a 2.0 kV applied electrospray voltage was used. The m/z scan was performed from 350 to 1,800, and it is extremely likely that at 70,000 resolutions, Orbitrap could find complete peptides. Following this, 28 peptides were chosen for MS/MS analysis using the NCE setting, and fragments with a resolution of 17,500 were found within Orbitrap. This data-dependent process alternated between one MS scan and 20 MS/MS scans with a dynamic exclusion time of 15.0 s. AGC was conFigd to be at 5E4. It was decided to use a primary attached mass of 100 m/z. The MaxQuant victimization search engine (v. 1.5.2.8) was used to process the generated MS/MS data. The reverse decoy information and the concatenated human UniProt information were compared in tandem mass spectra searches. Up to four mysterious cleavage events were made possible by the use of the cleavage protein trypsin/P. In the first search and the main search, the mass tolerance for precursor ions was set to 20 ppm and 5 ppm, respectively. The mass tolerance for fragment particles was set to 0.02 Da. Acetylation and oxidation of Met underwent a variety of changes. The minimum score for altered peptides was set at > 40, and FDR was adjusted to 1%.

Dissolution of tryptic peptides in NETN buffer (100 mM NaCl, 1 mM EDTA, 50 mM Tris-HCl, 0.5% NP-40, pH 8.0) to dissolve Kac-modified peptides. Prewashed antibody beads (PTM-104, PTM Bio) were then added and incubated with the peptide mixture at 4 °C overnight with gentle shaking. The beads were then washed twice with water and four times with NETN buffer. The use of 0.1% trifluoroacetic acid allowed the bound peptides to be released from the beads. The eluted portions were then mixed and dried under vacuum. The resultant peptides were desalted using C18 ZipTips (Millipore) in accordance with the manufacturer’s instructions for LC‒MS/MS analysis.

#### Bioinformatics analysis

First, a pair selection of samples to be compared was carried out, and the fold change (FC) was computed as a ratio of the average intensity at the different treatment locations in the two sets of samples. For example, to calculate the change in fold among samples CG and BG, the equation is given as follows: *R* is the relative quantitative value of the modification site, *i* is the sample, and *k* is the modification site.

$${FC}_{CG/BG,K}=Mean(R_{ik},i\in CG)/Mean(R_{ik},i\in BG)$$

To calculate the difference between the two groups, Student’s T test was conducted on the basis of the comparative quantitation of the change points between the BG and CG groups, and each P value was computed as the importance index. A *P* value < 0.05 was considered significant. For the data to be consistent with anormal distribution, the relative quantity of the modified site was log2 transformed. The equation is listed as follows:

$$P_k=T.test\;(Log2(R_{ik},i\in CG),Log2(R_{ik},i\in BG))$$

#### Subcellular localization

The cells of eukaryotic organisms are elaborately subdivided into functionally distinct membrane-bound compartments. Some major constituents of eukaryotic cells are extracellular space, cytoplasm, nucleus, mitochondria, Golgi apparatus, endoplasmic reticulum (ER), peroxisome, vacuoles, cytoskeleton, nucleoplasm, nucleolus, nuclear matrix and ribosomes. Here, we used WoLF PSORT subcellular localization prediction software to predict subcellular localization.

#### Analysis of KEGG pathway enrichment

To analyze the enrichment of KEGG pathways utilizing Kyoto Encyclopedia of Genes and Genomes (KEGG). The meanings of KEGG pathway enrichment (with identified proteins as background) for differential modification of proteins or genes were analyzed with Fisher’s exact test, and *P* values < 0.05 were considered significant.

#### Acetylated protein homology modeling

To identify the proteins, homologous sequences were searched using NCBI BLAST. SWISS-MODEL was used to build a 3D structural model of the protein and label the acetylation sites. Subsequently, protein hydrophobic clustering and salt bridge analysis were performed.

### Widely targeted metabolic study

#### Dry sample extraction

Using vacuum freeze-drying technology, the biological samples were placed in a lyophilizer (Scientz-100 F), and then the samples were ground (30 Hz, 1.5 min) to powder form by using a grinder (MM 400, Retsch). Next, 50 mg of sample powder was weighed using an electronic balance (MS105DΜ), and 1200 µL of -20 °C precooled 70% methanolic aqueous internal standard extract was added (less than 50 mg added at a rate of 1200 µL extractant per 50 mg sample). The mixture was vortexed once every 30 min for 30 s for a total of 6 times. After centrifugation (rotation speed 12,000 rpm, 3 min), the supernatant was aspirated, and the sample was filtered through a microporous membrane (0.22 μm pore size) and stored in the injection vial for UPLC‒MS/MS analysis.

#### UPLC conditions

A UPLC‒ESI‒MS/MS system (UPLC, ExionLC™ AD, https://sciex.com.cn/) and a tandem mass spectrometry system (https://sciex.com.cn/) were used to analyze the sample extracts. The analytical conditions were as follows: UPLC: column, Agilent SB-C18 (1.8 μm, 2.1 mm * 100 mm). The mobile phases were solvent A (pure water with 0.1% formic acid) and solvent B (acetonitrile with 0.1% formic acid). Sample measurements were carried out using a gradient procedure with starting conditions of 95% A and 5% B. Within 9 min, the gradient to 5% A and 95% B was increased and held for 1 min. Thereafter, the gradient was adjusted to 95% A and 5.0% B in 1.1 min and held for 2.9 min. The flow velocity was 0.35 mL/min. The column oven was set to 40 °C, and the injection volume was 2 µL. The effluent was alternatively linked to an ESI-triple quadrupole-linear ion trap (QTRAP)-MS.

#### ESI-Q TRAP-MS/MS

The ESI source operation parameters were as follows: ion spray voltage (IS) 5500 V (positive ion mode)/-4500 V (negative ion mode); source temperature 500 °C; ion source gas I (GSI), gas II (GSII), curtain gas (CUR), 50, 60, and 25 psi, respectively; collision-activated dissociation (CAD) was high. QQQ scans were obtained in the form of MRM experiments with nitrogen set to medium. DP (declustering potential) and CE (collision energy) for individual MRM shifts were performed with further DP and CE enhancement. A particular set of MRM transitions was monitored for each period according to the metabolites purged within the period of time.

#### Differentially abundant metabolites selected

For analyses in both the BG and CG groups, differentially abundant metabolites were determined by VIP (VIP > 1) and absolute Log2FC (|Log2FC| ≥ 1.5). VIP values were extracted from OPLS-DA results using the R package MetaboAnalystR, which also contains two plots, a score plot and replacement plots. The data were log-transformed and mean-centered before OPLS-DA. A permutation test (200 permutations) was performed to avoid overfitting.

## Result

### Decrease in photosynthesis, decrease in sucrose content and increase in starch content in* R. chrysanthum* under UV-B stress

**Fig. 2:**
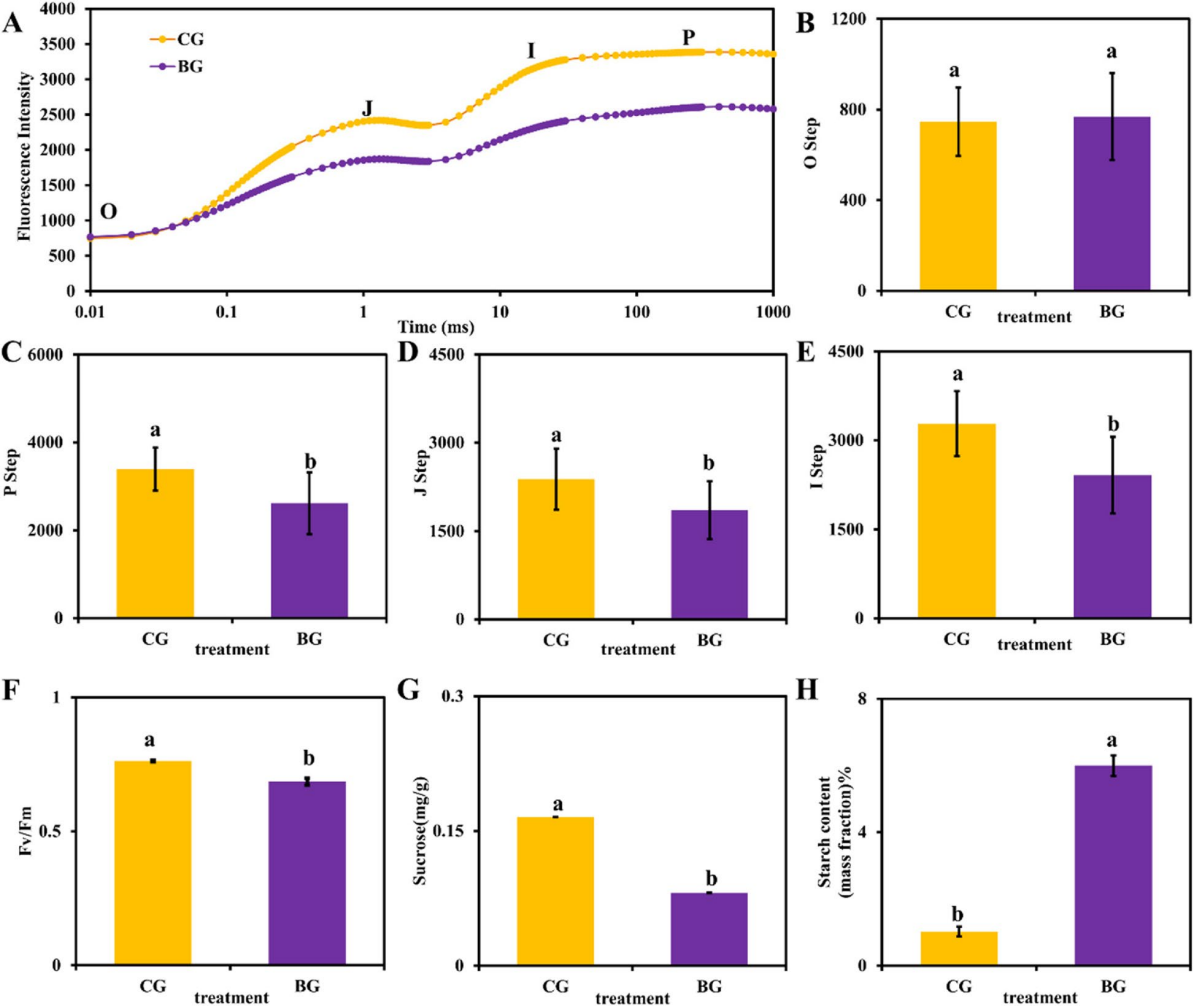
Effects of UV-B on chlorophyll fluorescence OJIP transient curves, starch and sucrose content in*R*. *chrysanthum* under UV-B stress. **A** Chlorophyll fluorescence OJIP transient curves. **B**-**E** Each step shown in the figure indicates the minimal fluorescence intensity when photosystem II (PSII) reaction centers (RCs) are open (the O step), the intensity at 2 ms (the J step), the intensity at 30 ms (the I step), and the maximal intensity when PSII RCs are closed (the P step). **F** Maximum photochemical efficiency (Fv/Fm). **G** Sucrose content. **H** Starch content. Bars represent SDs from three independent biological replicates (*n* = 3). Different letters indicate significant differences (*p* < 0.05). CG, PAR-treated *R. chrysanthum* plants; BG, PAR + UV-B treatment for 48 h

As shown in Fig. 2, the OJIP curves differed under UV-B stress, with UV-B stress leading to a decrease in fluorescence intensity, and the curves differed minimally at point O, and maximized at point I as electrons accumulated at points J and P. The OJIP curves differed at point I (Fig. 2B-E; Table 1). The JI phase was affected by the fast-reducing PQ library, and the IP phase was associated with the slow-reducing PQ library. The fluorescence intensities of the JI and IP phases were successively decreased in the BG group as compared to the CG group, which indicated that UV-B stress reduced the ability of the fast-reducing PQ library and the slow-reducing PQ library to be reduced, and the receptor side of the PSII reaction centers were injured. In *R. chrysanthum*, the leaf institutions were damaged under UV-B stress. In addition, the OJIP curves of the CG and BG groups were normalized (Supplementary Fig S1). The results showed that the relative variable fluorescence *V*_*J*_ at 2 ms in the *V*_*t*_ curve was significantly decreased under UV-B. The significant decrease in Fv/Fm of *R. chrysanthum* under UV-B stress indicated that UV-B stress caused photoinhibition and decreased photosynthesis in *R. chrysanthum* (Fig. 2F and Supplementary Table S1). The contents of sucrose and starch were closely related to photosynthesis, and the sucrose content decreased significantly and the starch content increased significantly under UV-B stress (Fig. 2G-H and Supplementary Table S1).

**Table 1:** Lists of acetylproteins involved in starch and sucrose metabolism in *R*. *chrysanthum* under UV-B stress

**Enzymes**	**Enzyme name**	**Protein accession**	**Modified position in protein**	**Modification sites fold change (BG/CG)**	**Protein expressed level (BG/CG)**
bglB	Beta-glucosidase	Gene.10123_CL754.Contig1_All	K206	Up	Down
GBSS	Granule-bound starch synthase	Gene.65666_Unigene6196_All	K156	Up	Up
SS	Soluble starch synthase	Gene.17918_CL1503.Contig2_All	K1013	Up	Up
bglX	beta-D-xylosidase	Gene.26794_CL2620.Contig8_All	K31	Up	Down
scrK	Fructokinase	Gene.27482_CL2732.Contig2_All	K169, K258	Up	Down
	Fructokinase	Gene.27485_CL2732.Contig3_All	K321, K272	Up	Down
	Fructokinase	Gene.46334_CL6624.Contig1_All	K65	Up	Down
malQ	4-alpha-glucanotransferase	Gene.32142_CL3482.Contig8_All	K551, K227	Up	Up
E3.2.1.21	Strictosidine-O-beta-D-glucosidase	Gene.33231_CL3688.Contig10_All	K525, K199, K275, K186	Up	Down
	Furcatin hydrolase	Gene.48469_CL7065.Contig1_All	K250, K444	Up	Down
GPI	Glucose-6-phosphate isomerase	Gene.4136_CL252.Contig3_All	K203	Up	Up
PGM	Phosphoglucomutase	Gene.53020_CL7992.Contig2_All	K269, K392	Up	Up
UGP2	UTP-glucose-1-phosphate uridylyltransferase	Gene.73617_Unigene12124_All	K247	Up	Up
glgC	Glucose-1-phosphate adenylyltransferase	Gene.79499_Unigene17572_All	K416	Up	Down
SUS	Sucrose synthase	Gene.8213_CL608.Contig1_All	K189, K586, K597	Up	Up

*Note*: Fold change (BG/CG) is the level of acetylated modification

### *R*. *chrysanthum* produces a large number of differential expressed proteins (DEPs) and differential expressed acetylated proteins (DAPs) under UV-B stress

To identify DAPs and DEPs of the *R. chrysanthum* plants under UV-B stress, we performed 4D label-free technology and acetylation modification analysis for both the BG and CG groups. In total, according to fold change > 1.5 and *p* < 0.05, 945 acetylation sites on 685 proteins were identified (Supplementary Fig. S2A and B, Supplementary Table S2). We identified 807 (450 upregulated and 357 downregulated) DEPs, 685 (95 upregulated and 590 downregulated) DAPs and 945 (104 upregulated and 841 downregulated) sites separately (Supplementary Fig S2C). We performed PCA to visualize differences between proteins and differences between metabolites, as well as differences between the two groups of samples. The results showed that the BG and CG groups, with three replicates clustered in each group, confirmed the reliability of the sample collection (Supplementary Fig S2D, E). The BG and CG were better separated, indicating differences between the different groups. Thus, many different expression levels of proteins were produced by the plants in the presence of UV-B.

### DEPs were mainly enriched in chloroplasts and starch and sucrose metabolic pathways of *R*. *chrysanthum* under UV-B stress

**Fig. 3:**
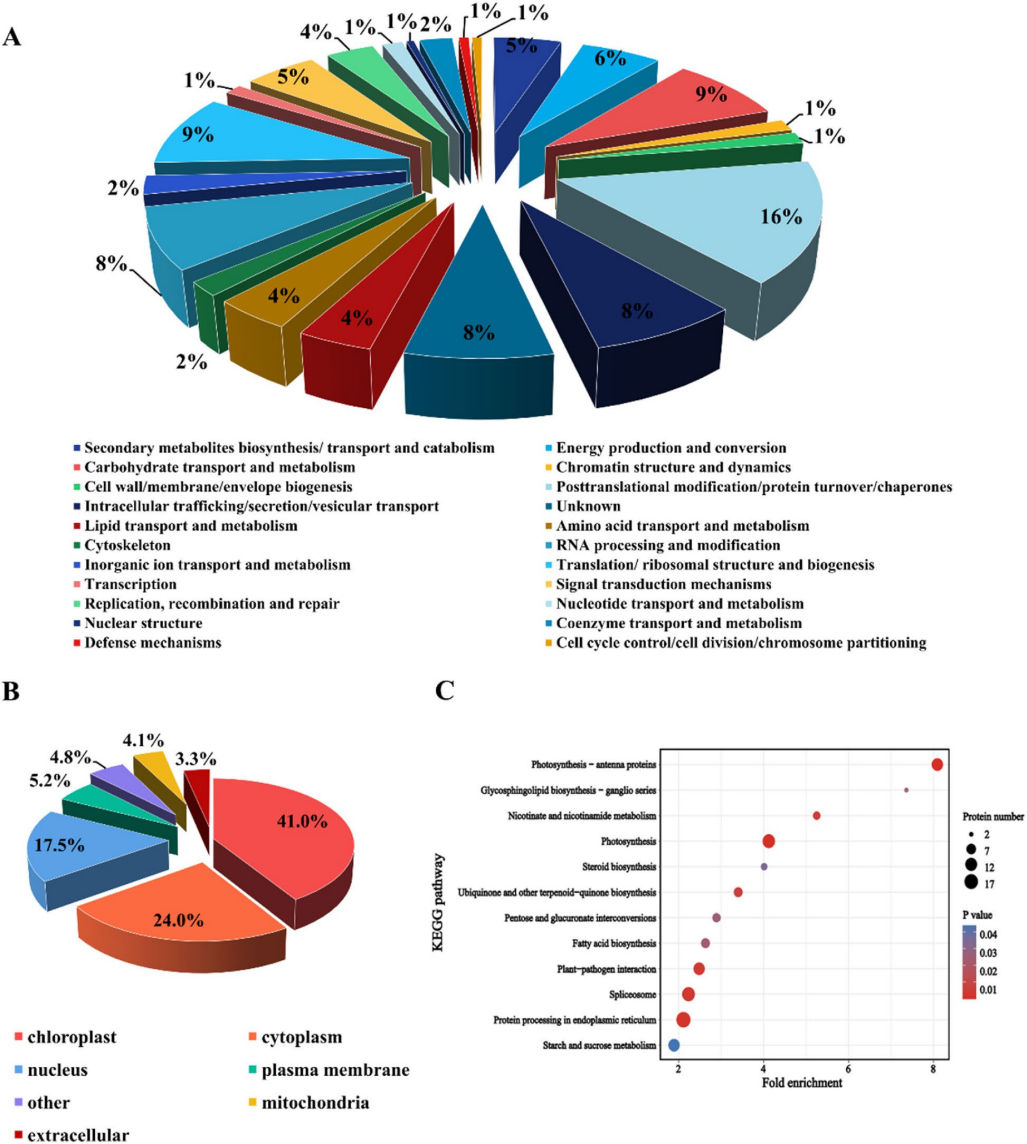
Functional classifications and enrichments of DEPs in *R*. chrysanthum under UV-B stress. **A** COG classifications of 807 DEPs; (**B**) Subcellular localization of DEPs; (**C**) KEGG enrichment of DEPs

According to the Clusters of Orthologous Groups (COG), the 807 DEPs were divided into twenty-two functional forms (Fig. 3A, Supplementary Table S3). These twenty-two functional protein categories were involved in posttranslational modification/protein turnover/chaperones (16%), defense mechanisms (1%), translation/ribosomal structure and biogenesis (9%), secondary metabolite biosynthesis/transport and catabolism (5%), carbohydrate transport and metabolism (9%), function unknown (8%), and RNA processing and modification (9%). This indicates that posttranslational modification of proteins and carbohydrate metabolism play an important role under UV-B stress. Subcellular localizations of the 807 confirmed proteins were anticipated by using WoLF PSORT (Supplementary Table S4). The results showed that 5.2% of DEPs were situated in the plasma membrane, 24.0% in the cytoplasm, 17.5% in the nucleus, 4.1% in the mitochondria, and 41.0% in the chloroplast, but 4.8% were other (Fig. 3B, Supplementary Table S4). This suggests that most of the proteins that are more affected by UV-B stress are concentrated in chloroplasts. Cluster analysis of KEGG pathway enrichment was used to investigate the pathways involved in DEPs of *R. chrysanthum* under UV-B stress. DEPs were obviously enriched in starch and sucrose metabolism, photosynthesis and other pathways under UV-B stress (Fig. 3C). The OJIP curves indicated that *R. chrysanthum* photosystem II was damaged under UV-B stress, affecting photosynthesis in the plant, which is related to the synthesis of starch and sucrose in the plant. Combined with the analysis of DEPs, this study focused on starch and sucrose metabolic pathways.

###  Acetylation modification of*R*. *chrysanthum* starch and sucrose metabolic pathway proteins under UV-B stress

**Fig. 4:**
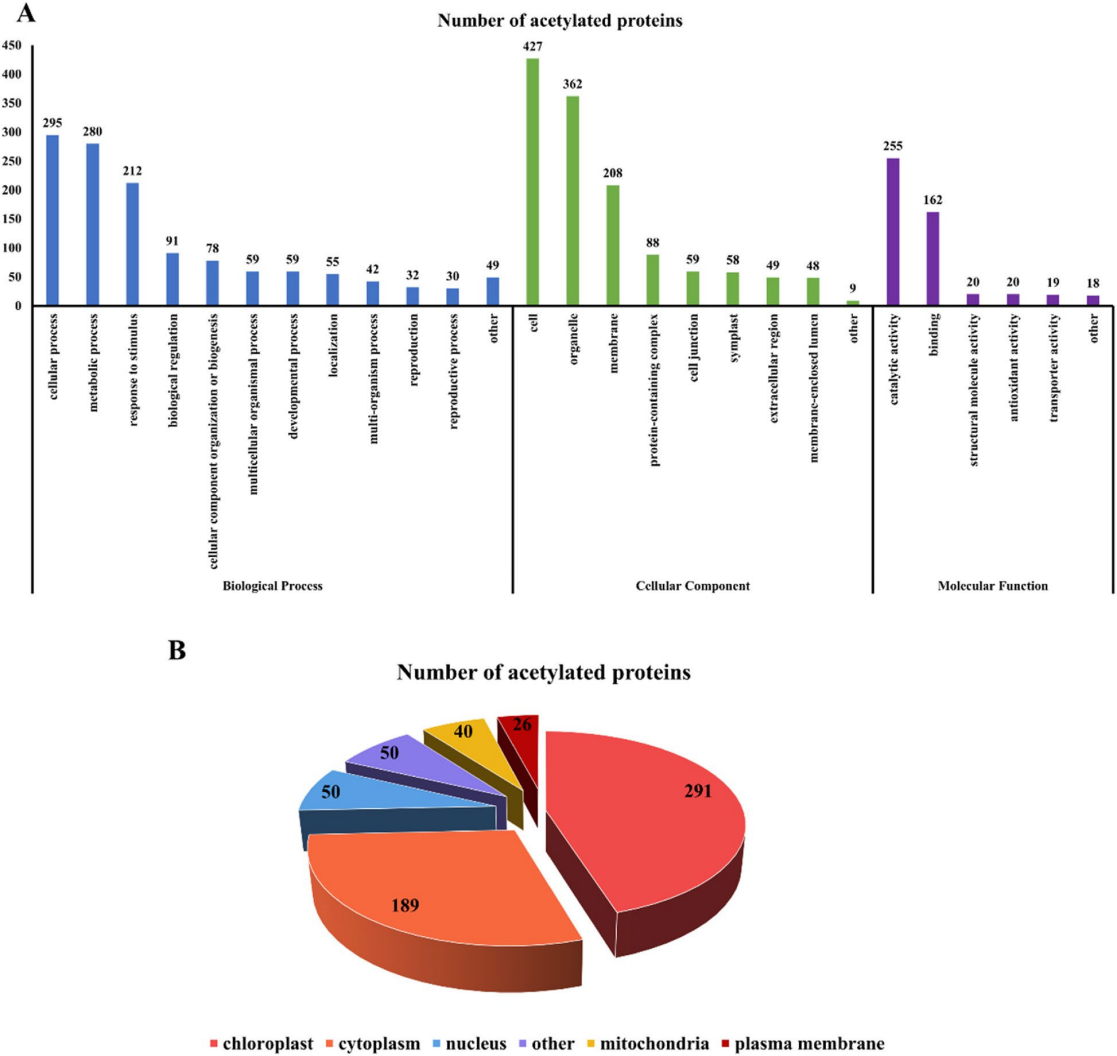
Functional classifications and enrichments of DAPs in *R*. *chrysanthum* under UV-B stress. **A** GO classifications of DAPs; (**B**) Subcellular localization of DAPs

In order to clarify the functions of DAPs, we performed GO function annotation and categorized DAPs into three main groups. In the category of biological processes, DAPs focused on cellular processes, metabolic processes and response to stimulus. In the category of molecular function, DAPs focused on catalytic activity and binding. These imply that enzymes occurring acetylation modifications play a significant role in *R. chrysanthum* resistance to UV-B stress (Fig. 4A). Subcellular localization analysis showed that DAPs were mainly concentrated in chloroplasts, followed by the cytoplasm. This is consistent with previous results of subcellular localization of DEPs. Indeed, starch synthesis occurs in chloroplasts and sucrose synthesis occurs in the cytoplasm. These imply that the enzymes involved in starch and sucrose metabolism undergo acetylation modification and play a role in UV-B resistance in *R. chrysanthum* (Fig. 4B). Based on the results of KEGG enrichment analysis of DEPs, we focused on acetylated proteins in the starch and sucrose metabolic pathways and identified a total of 15 DAPs and 25 acetylation modification sites. We found that most of the enzymes undergo acetylation modification during this metabolic process, and the modification levels were up-regulated (Table 1). It has been analyzed that in the starch and sucrose metabolic pathways, granule-bound starch synthase (GBSS), starch synthase (SS), 4-alpha-glucanotransferase (malQ), glucose-6-phosphate isomerase (GPI), phosphoglucomutase ( PGM), UTP-glucose-1-phosphate uridylyltransferase (UGP2), and sucrose synthase (SUS) were up-regulated at modification levels consistent with the protein expression levels, whereas the modification level of fructokinase (scrK), beta- glucosidase (bglB, bglX, E3.2.1.21) were inversely proportional to the expression level of the protein.

### Acetylation modification of proteins in the starch and sucrose metabolic pathways makes DEPs

**Fig. 5:**
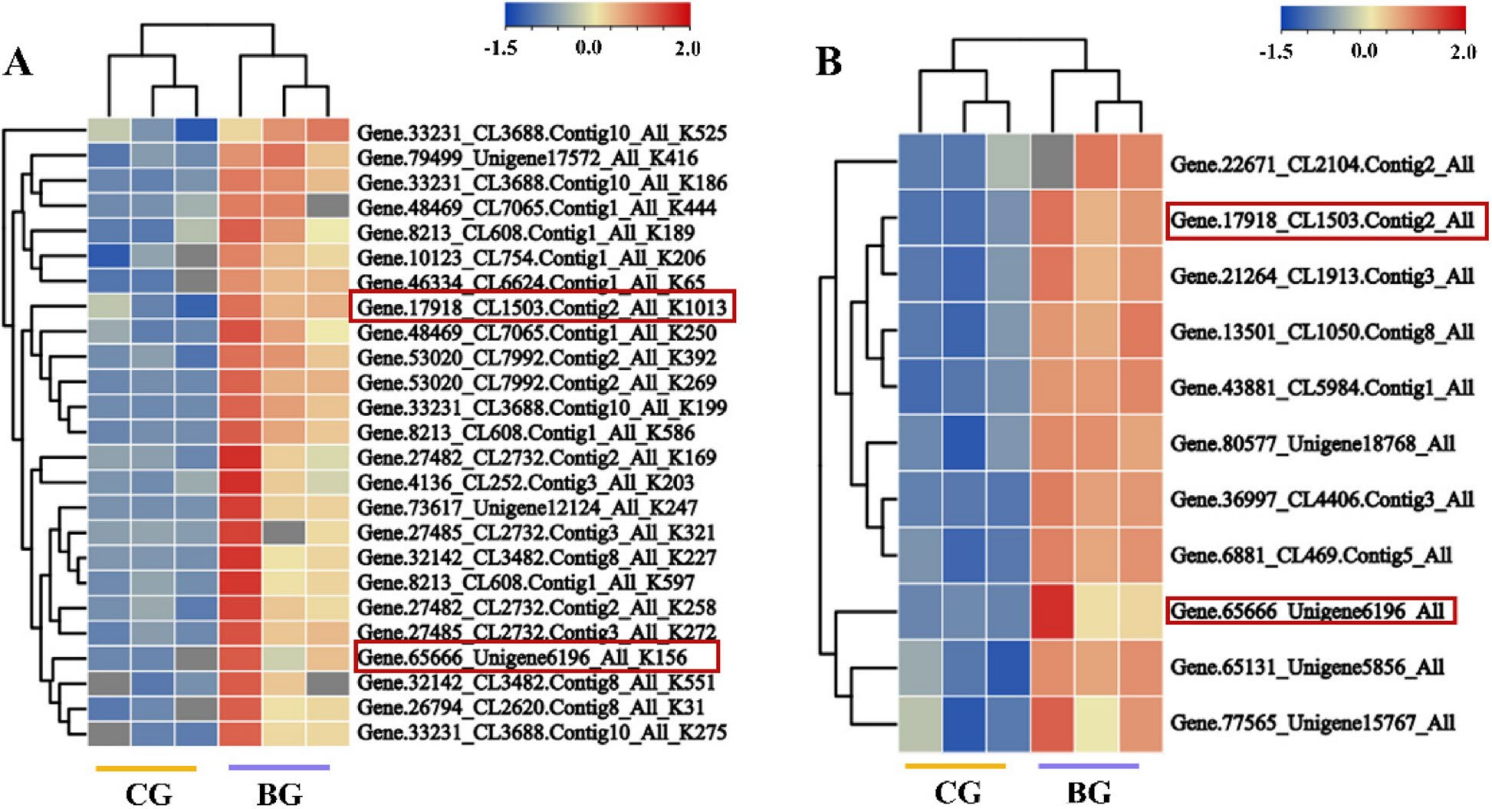
DEPs and DAPs of starch and sucrose metabolic pathways in *R*. *chrysanthum* under UV-B stress.** A** DAPs for starch and sucrose metabolic pathways. **B** DEPs for starch and sucrose metabolic pathways. Red boxes indicate DEPs undergoing acetylation modification

By analysis of acetylation modification histology, proteins of the starch and sucrose metabolic pathways of *R. chrysanthum* were modified by acetylation and up-regulated in level under UV-B stress (Fig. 5A). Proteomics analysis revealed that DEPs were significantly enriched in the starch and sucrose metabolic pathways, with a total of 11 DEPs, and the expression levels of the proteins were up-regulated (Fig. 5B). By comparative analysis, Gene.17918_CL1503.Contig2_All (SS) and Gene.65666_Unigene6196_ALL (GBSS) protein loci were found to undergo acetylation modification with significant differences in protein expression levels as DEPs. Therefore, we focused our analysis on SS and GBSS, which is a type of SS and a key enzyme in the synthesis of straight-chain starch. Thus, GBSS of *R. chrysanthum* plays an important role in resistance to UV-B stress.

### Noncovalent interactions of the proteins of pathway of sucrose and starch metabolism

**Fig. 6:**
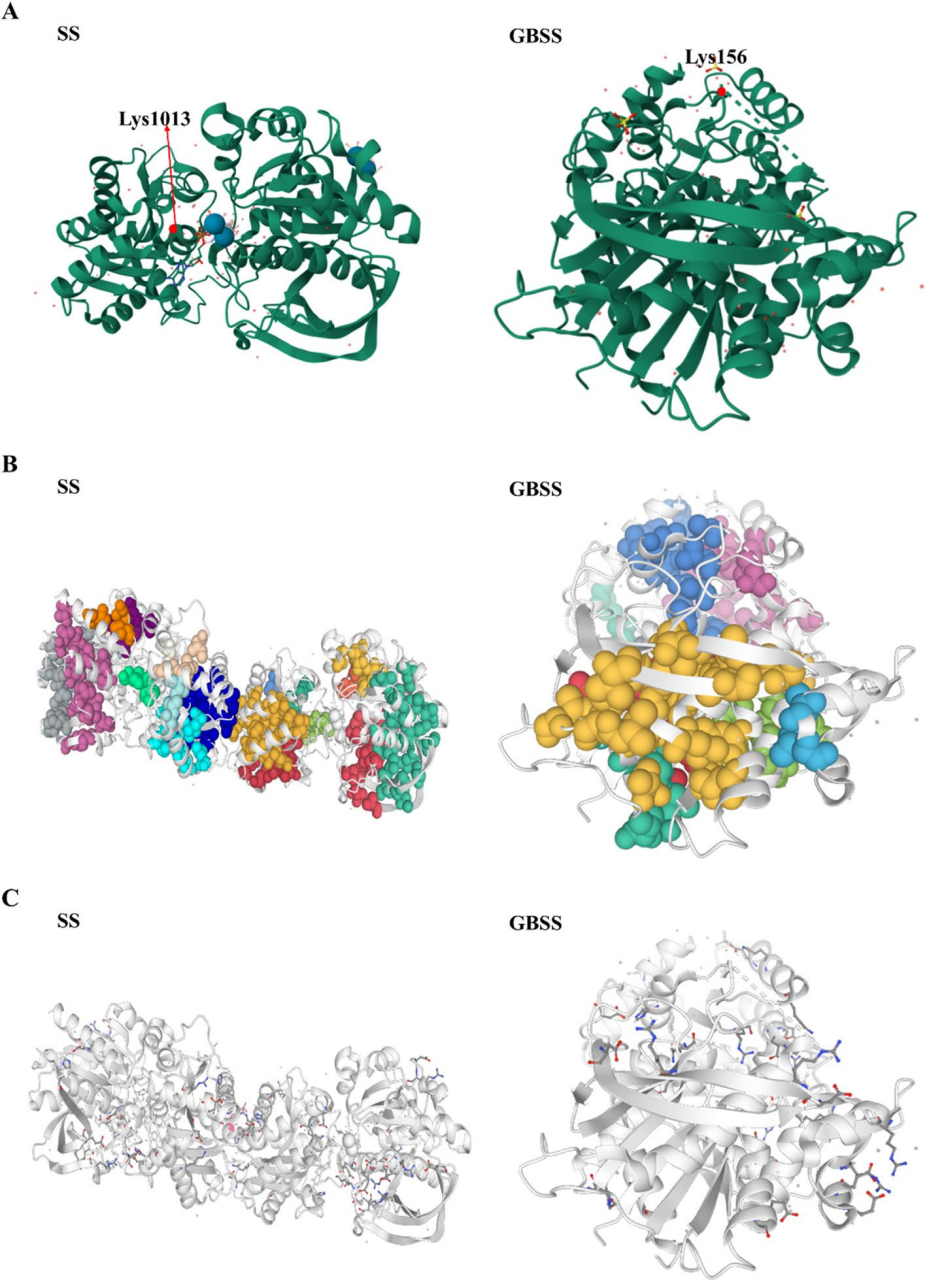
Three-dimensional structure construction of proteins undergoing acetylations in starch and sucrose metabolism pathways in *R. chrysanthum* under UV-B stress. **A** Three-dimensional structure, and the acetylation site is marked; (**B**) Hydrophobic cluster of the protein; (**C**) Salt-bridged structure of the protein. SS: starch synthase; GBSS: granule-bound starch synthase

**Fig. 7:**
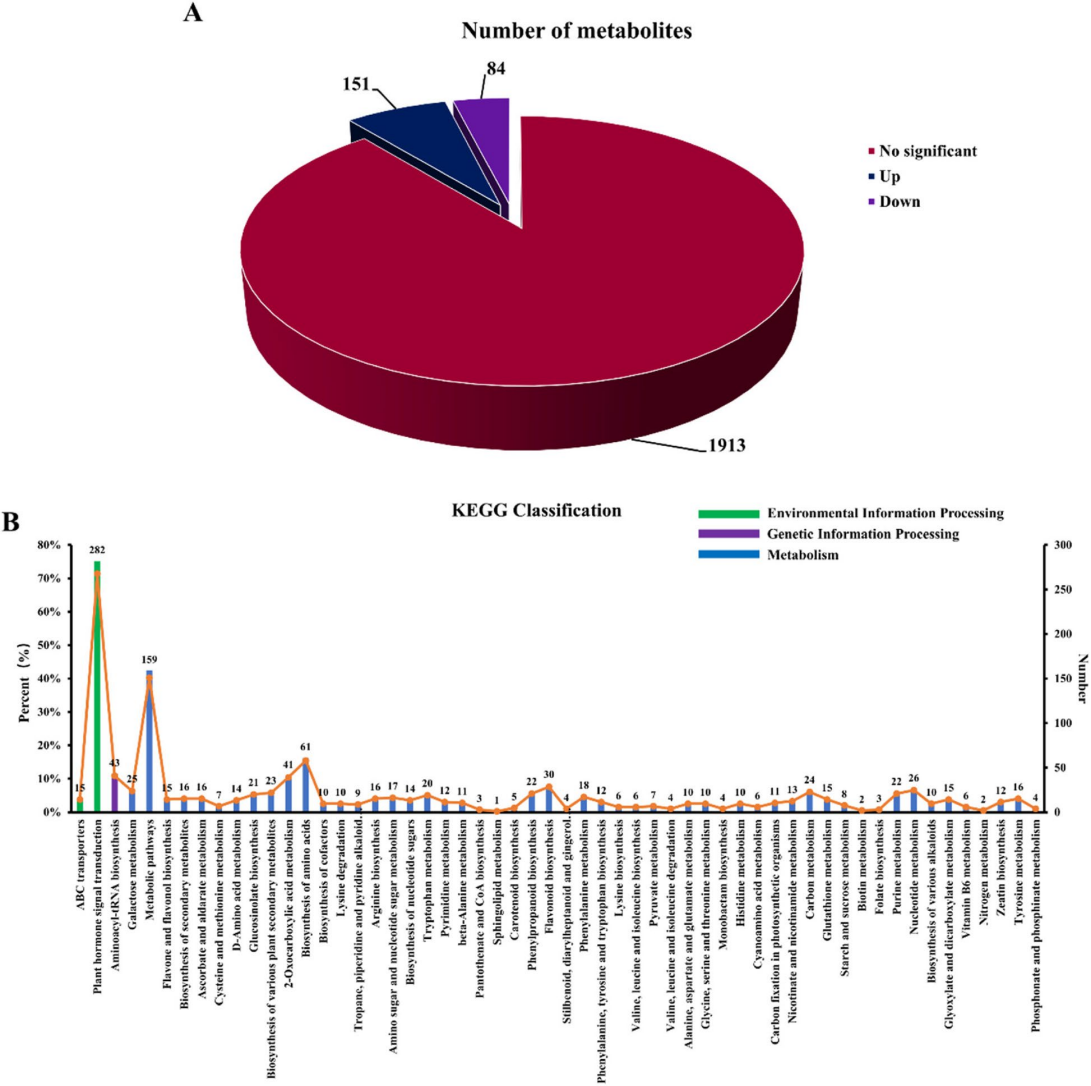
Metabolomic analysis of *R. chrysanthum* under UV-B stress. **A** Metabolite quantities. **B** KEGG classification of differential metabolites

To better understand protein acetylations during UV-B stress, we constructed models of acetylated proteins in starch and sucrose metabolism. SWISS-MODEL was used to create statistically acceptable homology models, labeled acetylation sites in the three-dimensional model and performed protein interaction structural analysis (Fig. 6). Proteins fold into their natural structures driven by various noncovalent interactions (hydrophobic and ionic interactions). Therefore, a characterization of these interactions is necessary to understand protein features and functions at the molecular level. We analyzed the SS and GBSS enzymes and represented their acetylated modification in the three-dimensional structures of the proteins (Fig. 7A). SS, significant difference in acetylation modification site was Lys1013. GBSS, significant difference in acetylation modification site was Lys156. By analyzing the hydrophobic clusters, SS showed that the largest cluster contained 21 residues with a total area of 3158.9^2^. GBSS showed that the largest cluster contained 16 residues with a total area of 2277^2^ (Fig. 7B, Supplementary Table S5). Salt bridges contribute to the function and stability of proteins, and the composition of band-point side chains distinguishes the folding mechanism of proteins from that of natural proteins. The (Fraction of Charged Residues) FCR of SS was analyzed as 0.23, Kappa value (k) was 0.16. And the FCR of GBSS was analyzed as 0.24, k was 0.15 (Fig. 7C).

#### Metabolomic analysis of *R. chrysanthum* under UV-B stress

A total of 2148 metabolites were detected by broadly targeted metabolomics assays, of which 235 metabolites were identified as differential metabolites (DAMs), 151 were upregulated and 84 were downregulated (Fig. 6A). In order to clarify the functions of DAMs, KEGG classification analysis of DAMs was performed. DAMs were classified into three main categories, focusing mainly on metabolism and, to a lesser extent, environmental information processing. Among them, DAMs were also concentrated in starch and sucrose metabolism, which was consistent with the proteome (Fig. 6B). This suggests that starch and sucrose metabolism play a role in UV-B stress resistance in *R. chrysanthum*.

### Metabolomic and acetylated proteomic analysis of starch and sucrose metabolism in *R. Chrysanthum*

**Fig. 8:**
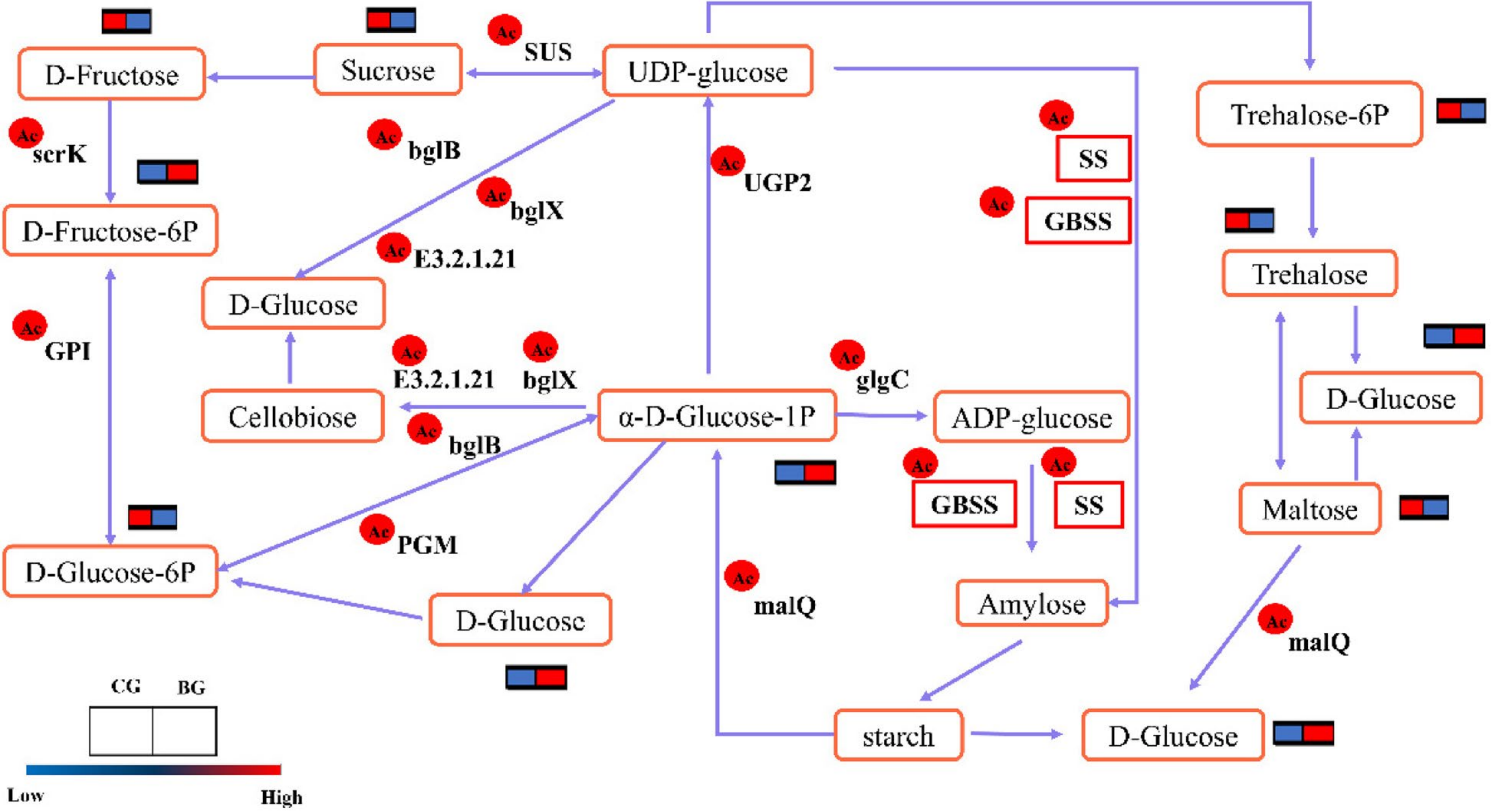
Changes in key metabolites in starch and sucrose metabolic pathways of *R. chrysanthum* under UV-B stress. The red circle in the upper left corner indicates the upregulation of the level of acetylation modification of the enzymes, and the clustered heatmap indicates the change in metabolites, from blue to red indicating low to high levels

On the basis of analyzing the changes in the expression and acetylation levels of proteins of the sucrose and starch metabolic pathways, we explored the changes in the expression of metabolites during starch and sucrose metabolism in *R. chrysanthum* under UV-B stress. From Fig. 8, it can be seen that there were differences in the expression of key metabolites of starch and sucrose pathways when *R. chrysanthum* was subjected to UV-B stress. In the starch synthesis and catabolism pathway, the content of α-D-glucose-1-phosphate metabolites was upregulated. In the sucrose synthesis and metabolism pathway, the content of trehalose-6P, trehalose, sucrose, and D-fructose was downregulated. The decrease in sucrose content and the increase in starch content were analyzed by combining the experimental results with the results of protein acetylation. The results showed that the content of *R. chrysanthum* the acetylation of GBSS promoted the synthesis of starch, and the sucrose content decreased.

## Discussion

Starch and sucrose metabolism is important for plant development and for tolerating environmental stresses [14, 16, 24, 44]. *R. chrysanthum* plants readjust carbohydrate contents and related enzyme-encoding gene expression when exposed to UV-B stress [38]. The dynamics of these changes suggest that carbohydrate partitioning and utilization under UV-B stress are closely related to plant UV-B tolerance. Based on the previous studies on photosystem proteins, this study takes the starch and sucrose metabolic pathways associated with photosynthesis as the research target, and the acetylation of key enzymes in the metabolic pathways to investigate the effect of acetylation on carbohydrate expression in plants under UV-B stress and to reveal the molecular mechanism of UV-B resistance in plants.

Leaves are the major photosynthetic organs of *R. chrysanthum*, but recent studies have shown that photosynthesis in *R. chrysanthum* is inhibited and the photosystem is impaired under UV-B stress [22]. In previous studies, we found that the *R. chrysanthum* photosystem II protein undergoes acetylation modification in response to UV-B stress. Previous studies have focused on photosynthesis in plants, and have not studied the role of protein acetylation in photosynthesis-related pathways. In fact, photosynthesis is closely related to the metabolism of starch and sucrose. Triose phosphate, a product of photosynthesis, is a substrate for starch and sucrose synthesis, and inhibition of photosynthesis affects starch and sucrose synthesis. It was reported that high concentrations of CO_2_ reduce the content of starch and sucrose in Goji berry (*Lycium barbarum* L.) [26]. The levels of protein, sugar and starch in maize seeds decreased with enhanced UV-B radiation, whereas the level of lysine increased with enhanced UV-B radiation [12]. In studies on *Pelargonium zonale*, it was also clearly shown that UV-B stress stimulates the breakdown of starch and sucrose and affects sugar partitioning and utilization [41]. In our research, in the starch synthesis and catabolism pathway, the content of metabolites was upregulated, such as α-D-glucose-1P and D-Glucose, but maltose was downregulated, while in the sucrose synthesis and catabolism pathway, the content of Trehalose-6P, Trehalose, sucrose, and D-Fructose was downregulated (Fig. 8).

The strength of substance metabolism is regulated by enzymes that affect changes in the content of the responding substances [42]. Proteins are altered by UV-B radiation, so UV-B radiation is closely related to enzyme activity, which directly affects changes in metabolites. It has been reported that the expression of genes regulating starch and sucrose metabolic pathways in *Aquilegia vulgaris* under salt stress varies with the salt treatment process [5]. In rice, the content of carbohydrates, especially sucrose, decreased during UV-B exposure, and the expression of genes in the generation pathway varied with the intensity of UV-B radiation [43]. We clearly recognize the effects of UV-B stress on key enzymes of starch and sucrose metabolism in plants, which in turn affects metabolite content. However, no study has analyzed the acetylation of key enzymes in starch and sucrose metabolic pathways in plants under adverse stress and the acetylation effect on the expression of carbohydrates, such as starch and sucrose. In this study, we revealed the response of the *R. chrysanthum* starch and sucrose pathways to UV-B stress through acetylation proteomics and widely targeted metabolomics analyses and found that in this pathway, some enzymes are modified by acetylation under the influence of UV-B, whereas them undergo changes in the level of acetylation under the influence of UV-B stress, which in turn alters the expression of proteins and affects metabolite expression.

**Fig. 9:**
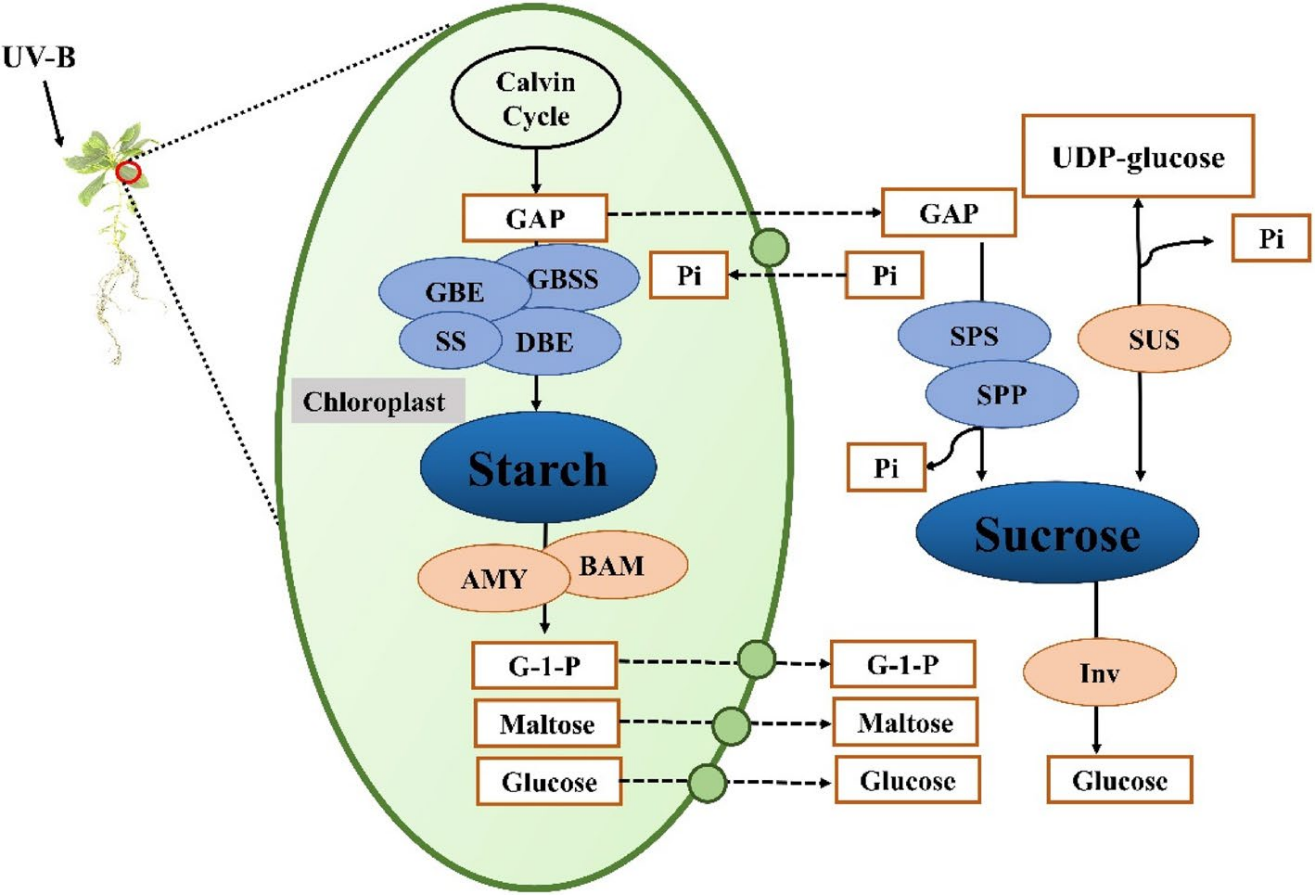
Starch and sucrose metabolism in *R. chrysanthum *under UV-B stress. SPP: sucrose phosphatase; SPS: sucrose-phosphate synthase; Inv: invertase; GBE: 1,4-alpha-glucan branching enzyme; SS: starch synthase; GBSS: granule-bound starch synthase; AMY: alpha-amylase; BAM: beta-amylase; DBE: starch debranching enzyme. Metabolites and biomolecules are shown in boxes; enzymes are shown in ovals; dotted lines indicate membrane transport; circles indicate transport of substances across membranes

In fact, impaired photosynthesis in plants under UV-B stress affects the ability of the plant body to fix carbon, which in turn affects starch and sucrose metabolism (Fig. 2). The plant body uses triose phosphate as a common substrate and converts it into two major carbohydrates, sucrose synthesized in the cytoplasm and starch synthesized in the chloroplast. On the one hand, in chloroplasts, triose phosphate is used to synthesize starch by enzymes, including GBE, GBSS, DBE, and SS. Starch undergoes hydrolysis through the action of AMY and BAM enzymes [13, 28]. On the other hand, triose phosphate undergoes sucrose synthesis in the cytoplasm and produces sucrose in the activities of SPP and SPS, and there are two pathways of sucrose catabolism. Sucrose is decomposed to fructose, UDP-glucose and inorganic phosphate by the action of SUS. The other pathway of sucrose hydrolysis is the hydrolysis of sucrose into fructose and glucose using sucrase, which is later taken up by the cells [33, 35] (Fig. 9). In our research, Under UV-B stress, the content of starch increased in *R. chrysanthum*. Our data further demonstrate that acetylations of these starch and sucrose metabolism-related proteins play an important role in resistance to UV-B stress in *R. chrysanthum* plants.

## Conclusion

We performed comprehensive analyses of *R. chrysanthum* biochemistry and acetylation proteomics and extensive targeted metabolomics under 48 h PAR and 48 h PAR + UV-B stress. The experimental results showed that the OJIP curves represent the receptor side of the PSII reaction center, *R. chrysanthum* sucrose content decreased and starch content increased under UV-B stress. KEGG enrichment analysis showed that starch and sucrose metabolic pathways and photosynthesis pathways were significantly enriched in *R. chrysanthum*. Acetylated proteome revealed that enzymes in the sucrose and starch metabolic pathways underwent acetylation upon UV-B stress. Further analysis revealed that GBSS undergoes acetylation modification and the level of modification is up-regulated, and this enzyme is DEP in the starch and sucrose pathways, implying that this enzyme has an important role in UV-B resistance in *R. chrysanthum*. Combined the acetylated proteome and broadly targeted metabolomics analyses showed that the level of acetylation modification of *R. chrysanthum*. GBSS was up-regulated under UV-B stress, and the expression of proteins was also significantly up-regulated, which promoted starch synthesis and elevated starch content. Therefore, our results suggest that photosynthesis of *R. chrysanthum* decreased under UV-B stress, affecting metabolic processes. acetylation modification and up-regulation of the level of GBSS occurred, and protein expression was also significantly up-regulated, which promoted the synthesis of starch and elevated the starch content, making *R. chrysanthum* resistant to UV-B stress.

### Supplementary Information

**Supplementary Material 1.**

**Supplementary Material 2.**

**Supplementary Material 3.**

**Supplementary Material 4.**

**Supplementary Material 5.**

**Supplementary Material 6.**

## Data Availability

The datasets analysed during the current study are available in the iProX repository, https://www.iprox.cn/page/project.html?id=IPX0007178000 or PXD046044.
