# Supplementary material for: Acetylation proteomics and metabolomics analyses reveal the involvement of starch synthase undergoing acetylation modification during UV-B stress resistance in Rhododendron Chrysanthum Pall
Source: Hereditas. 2024 May 3;161:15. doi: 10.1186/s41065-024-00320-4 (PMC11067277; doi:10.1186/s41065-024-00320-4)
Supplement: Supplementary file 1 — Supplementary Material 1. [file 41065_2024_320_MOESM1_ESM.docx]

[Molecular Breeding](https://www.springer.com/journal/11032)

**Acetylation proteomics and metabolomics analyses reveal the involvement of starch synthase undergoing acetylation modification during UV-B stress resistance in *Rhododendron chrysanthum* Pall.**

Meiqi Liu^1^, Li Sun^2^, Yuhang Cao^1^, Hongwei Xu^1^ and Xiaofu Zhou^1, *^

^1^Jilin Provincial Key Laboratory of Plant Resource Science and Green Production, Jilin Normal University, Siping, China.

^2^ Siping Central People’s Hospital, Siping, China.

*** Correspondence:** zhouxiaofu@jlnu.edu.cn.

# Supplementary Figures and Tables

## Supplementary Figures


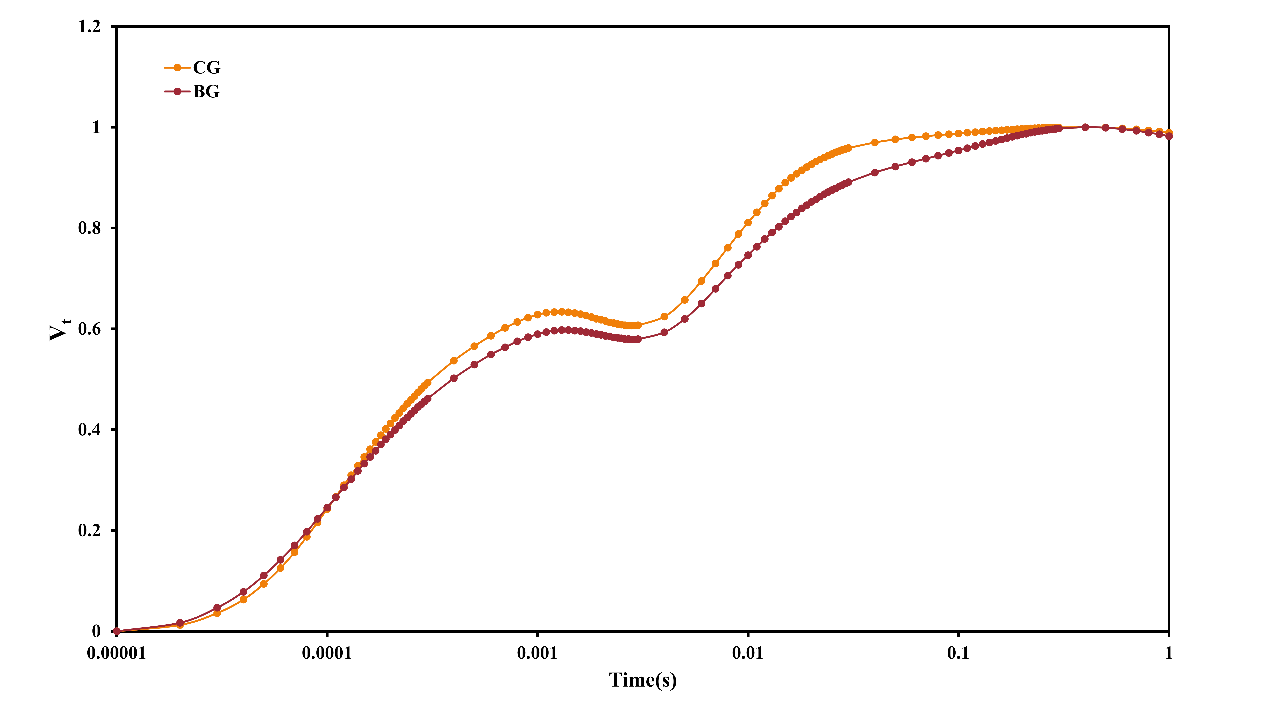


**FIGURE S1.** Effects of *Vt* curves in *R. chrysanthum* under UV-B stress. The data in the figure are from three replicated experiments (*n* = 3). CG, PAR treatment *R. chrysanthum* plants; BG, PAR+UV-B treatment for 48 h.


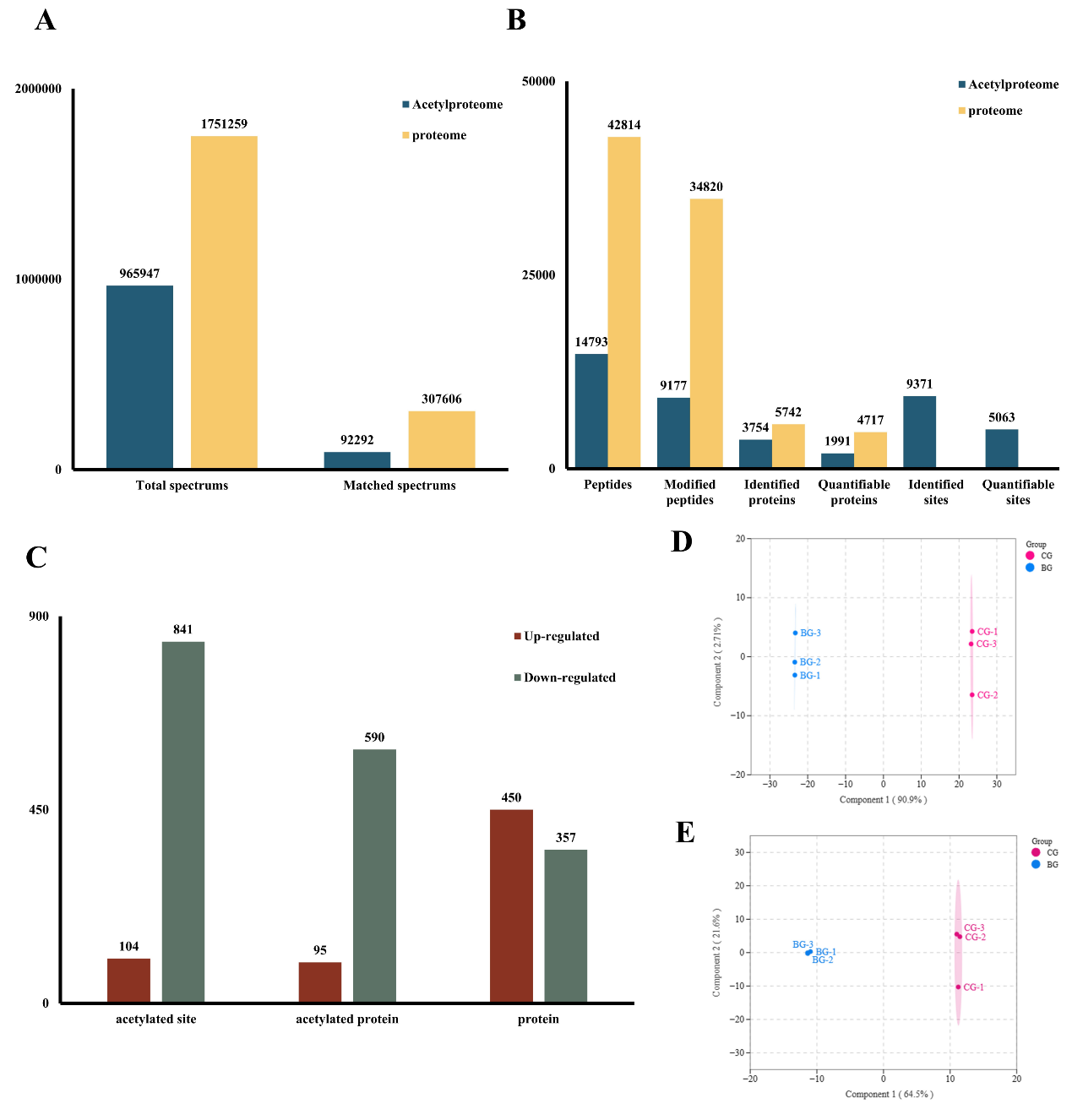


**FIGURE S2.** Basic information on acetylated proteome, proteome and metabolome analysis. (A-B) Analysis of proteomic and acetylproteome data; (C) The number of DAPs and DEPs; (D) PCA analysis based on proteomic data; (E) PCA analysis based on metabolomic data.
